# Calculation and Visualization of Binding Equilibria in Protein Studies

**DOI:** 10.1021/acsomega.2c00560

**Published:** 2022-03-16

**Authors:** Johan Pääkkönen, Janne Jänis, Juha Rouvinen

**Affiliations:** Department of Chemistry, University of Eastern Finland, P.O. Box 111, 80101 Joensuu, Finland

## Abstract

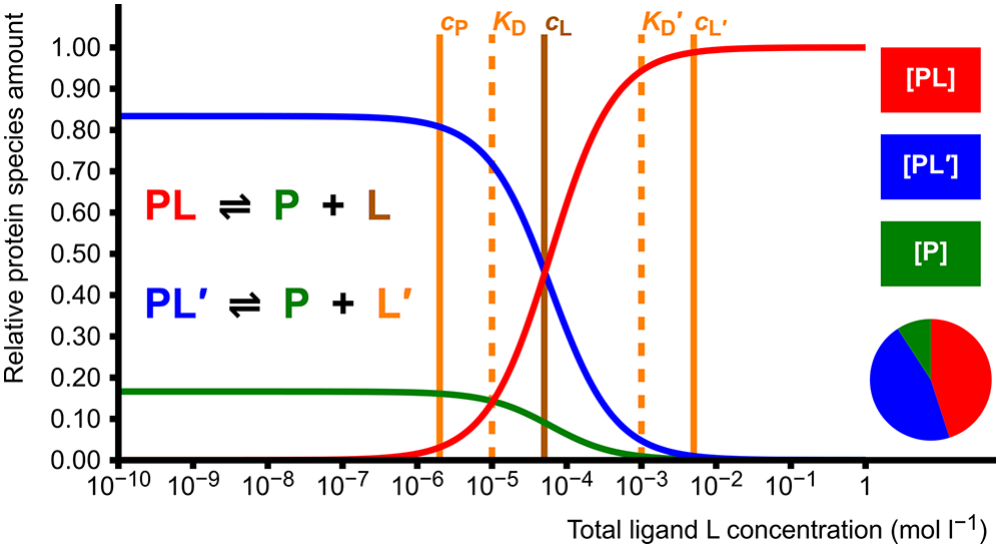

A set of simulation applets has been developed for visualizing the behavior of the association and dissociation reactions in protein studies. These reactions are simple equilibrium reactions, and the equilibrium constants, most often dissociation constant *K*_D_, are useful measures of affinity. Equilibria, even in simple systems, may not behave intuitively, which can cause misconceptions and mistakes. These applets can be utilized for planning experiments, for verifying experimental results, and for visualization of the equilibria in education. The considered reactions include protein homodimerization, ligand binding to a receptor (or heterodimerization), and competitive ligand binding. The latter one can be considered as either a ligand binding to two receptors or a binding of two ligands to a single receptor. In general, the user is required to input the total concentrations of all proteins and ligands and the dissociation constants of all complexes, and the applets output the equilibrium concentrations of all protein species graphically as functions of concentration and as numerical values at a specified point. Also, a curve fitting tool is provided which roughly estimates the concentrations or the dissociation constants based on the experimental data. The applets are freely available online (URL: https://protsim.github.io/protsim) and readily hackable for custom purposes if necessary.

## 1 Introduction

Biomolecular complex formation—the association of protein with other proteins or ligands as well as self-association—is an essential property of protein function. Association can be expressed with simple mathematical models describing chemical equilibria which were originally based on the law of mass action. The history for these models is long: Hüfner applied already in 1890 a chemical equilibrium model for describing dioxygen binding to hemoglobin.^1^ The equilibrium model can be utilized successfully in many kinds of systems describing protein association. Amounts of different species in equilibrium can be calculated if the total concentrations of components and equilibrium constants (usually dissociation constants *K*_D_) are known. However, the calculations become complicated when the number of system components increases, hampering the use of equilibrium models in protein studies.

Understanding reaction thermodynamics is an important skill for all chemists. Equilibrium states do not always behave intuitively when the conditions are changed, and people sometimes misinterpret their data and draw incorrect conclusions. Especially when working with reaction equilibria, for instance with proteins, ligands, and protein complexes, knowing the thermodynamic laws and their consequences is crucial. This knowledge helps with both planning experiments and interpreting results obtained therefrom.

In this work, a set of simulation applets which can be used to simulate complex formation of proteins has been developed. These tools can be used in designing experiments and interpreting results concerning the association behavior of proteins as well as for educational purposes. Visualization of equilibrium concentrations is especially useful in getting a quick grasp of how the concentrations behave when conditions are altered. The tools presented in this article allow such visualizations with simple controls and illustrative graphics.

Good planning of experiments saves time and materials. It is wasteful to prepare and measure samples that give no useful information, for instance, if one species is so dominant that others cannot be seen. In the typical case of figuring out dissociation constants, if a rough estimate of the values can be given, suitable conditions to measure can be determined, but it is not trivial. The simulation applets allow that by showing how the equilibrium concentrations behave when the initial concentrations are input.

Few examples of similar previously published work exist. Shave et al.^2^ have developed a Python package with which homo- and heterodimerization inhibition can be modeled and similar graphs can be generated. While powerful, it requires that the user be able to write program code, the learning curve of which is generally prohibitive. The applets presented in this paper have purposefully been made simplistic so that anyone could use them with ease without needing to learn any programming skills. Regardless, a sufficiently skilled programmer could customize the applets for any needs not already covered.

## 2 Theory

### 2.1 Definitions

For a general association reaction
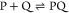
1the equilibrium constant is called the association constant *K*_A_ and is defined as
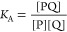
2The inverse of this, the dissociation constant *K*_D_, has the same unit as the concentration, which makes it useful since it has a physically and chemically meaningful value:
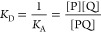
3The Gibbs free energy of the reaction Δ*G* is defined as
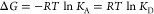
4where *R* is the molar gas constant (∼8.314 J K^–1^ mol^–1^) and *T* is temperature (typically 298.15 K = 25 °C). The logarithm is defined only for dimensionless numbers, and in this case *K*_D_ always has a concentration unit. However, the convention is to effectively take the concentration value in moles per liter and to discard the unit.^3^

### 2.2 Homodimerization

**Figure 1:**
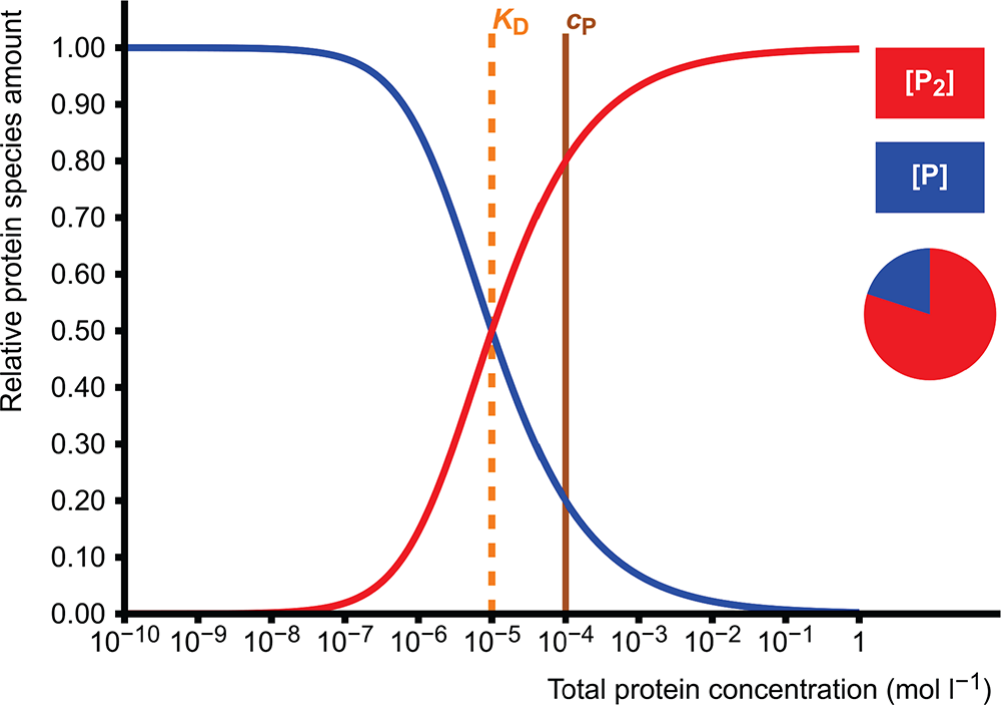
Dimer formation as a function of total protein concentration *c*_P_. The pie diagram depicts the proportions of the dimeric (P_2_, red, 80.0%) and monomeric (P, blue, 20.0%) forms at the set value of *c*_P_ = 1.0 × 10^–4^ mol L^–1^. The dissociation constant *K*_D_ is set as 1.0 × 10^–5^ mol L^–1^, and it corresponds to the total protein concentration where the protein is half (50%) dimerized.

Homodimerization or self-association of protein monomers is described by an equilibrium between dimer (P_2_) and monomer (P):
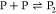
5There are two adjustable parameters in the model: one total concentration and one dissociation constant *K*_D_, which results in a simple quadratic equation (Supporting Information, section 1.2). Equilibrium concentrations of free monomer P and dimer P_2_ are calculated. This model is useful especially if the protein forms a weak or transient dimer with relatively high *K*_D_,^4^ in which case the amount of dimer is highly dependent on the protein concentration used in experiments (Figure 1). On the other hand, the model can be used to estimate an approximate *K*_D_ if the amounts of monomer and dimer in equilibrium are known.

### 2.3 Ligand Binding to a Receptor

In this case, there is an equilibrium between the protein–ligand complex (PL) and the free uncomplexed species (P and L):
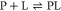
6

**Figure 2:**
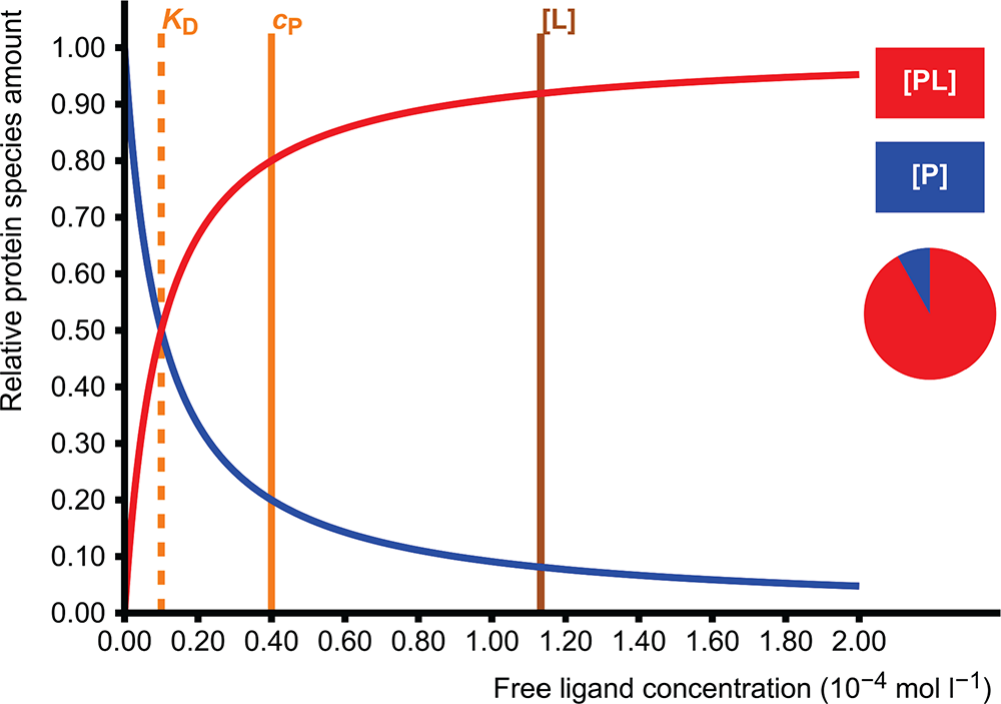
Linear ligand binding isotherm shows the ligand bound fraction of protein as a function of free ligand concentration [L]. The pie diagram depicts the proportions of the complex (PL, red, 90.8%) and free protein (P, blue, 9.2%) at the free ligand concentration which results from total protein concentration *c*_P_ = 4.0 × 10^–5^ mol L^–1^, total ligand concentration *c*_L_ = 1.5 × 10^–4^ mol L^–1^ and dissociation constant *K*_D_ = 1.0 × 10^–5^ mol L^–1^. In this representation, the dissociation constant corresponds to the free ligand concentration in which the protein is half (50%) saturated.

There are three adjustable parameters in this model: two total concentrations and one dissociation constant *K*_D_, which again results in a quadratic equation (Supporting Information, section 2.2). Equilibrium concentrations of PL, P, and L are calculated, though only the species containing protein P are shown in the visualization. This model can be used to describe many kinds of important associations such as drug binding to a receptor, metal binding to a binding site,^5^ substrate or inhibitor binding to an enzyme, and protein heterodimerization (Figure 2).

### 2.4 Competitive Binding of Two Ligands to One Receptor

In this model, two different ligands L and L′ compete with each other in binding to the same site of a receptor protein P.
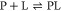
7
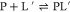
8

**Figure 3:**
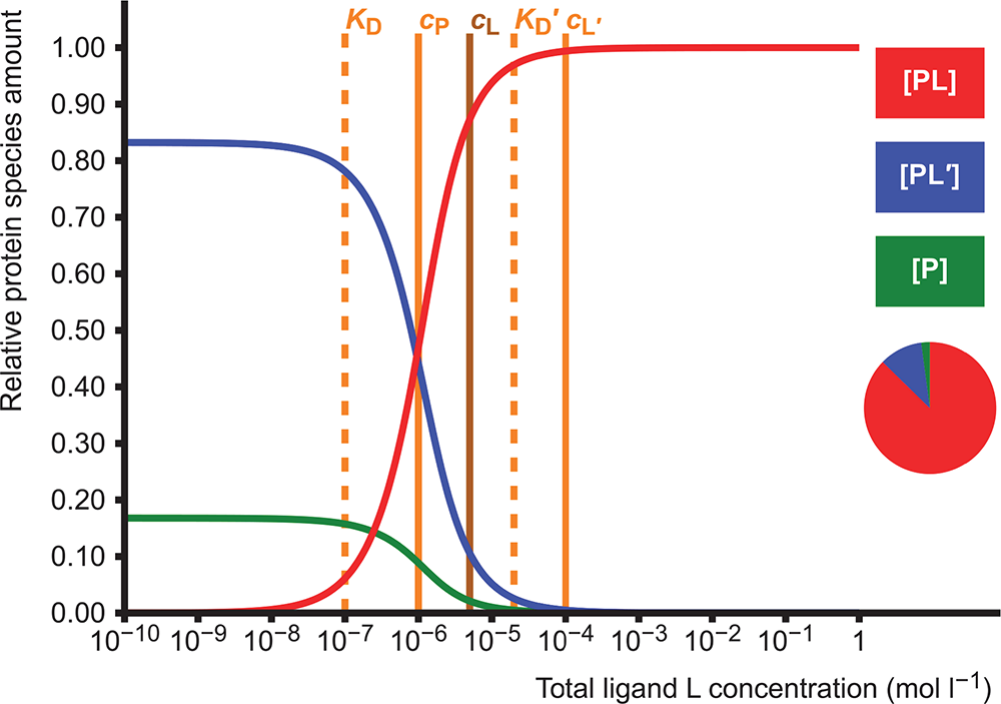
Receptor protein occupancies as functions of the total concentration of competing ligand L. The pie diagram depicts the proportions of the complexes PL (red, 87.3%) and PL′ (blue, 10.6%) and the free protein P (green, 2.1%) at the set value of *c*_L_ = 5.0 × 10^–6^ mol L^–1^. In this case, the concentration of receptor protein P is *c*_P_ = 1.0 × 10^–6^ mol L^–1^, and the concentration of reference ligand L′ is *c*_L′_ = 1.0 × 10^–4^ mol L^–1^. The dissociation constants are *K*_D_ = 1.0 × 10^–7^ mol L^–1^ for the PL complex and *K*_D_^′^ = 2.0 × 10^–5^ mol L^–1^ for the PL′ complex.

There are five adjustable parameters in the model: three total concentrations (for protein P and two ligands L and L′) and dissociation constants (*K*_D_ and *K*_D_^′^) for two complexes. This produces a cubic equation which is solved numerically, and equilibrium concentrations of all PL, PL′, P, L, and L′ are calculated. Receptor occupancy is calculated as a function of either total protein concentration or total ligand L concentration (Figure 3). The latter one is useful in designing and analysis of, for example, radioligand displacement or inhibition curves which can be further used to estimate IC_50_ values.

### 2.5 Competitive Binding of a Ligand to Two Receptors

**Figure 4:**
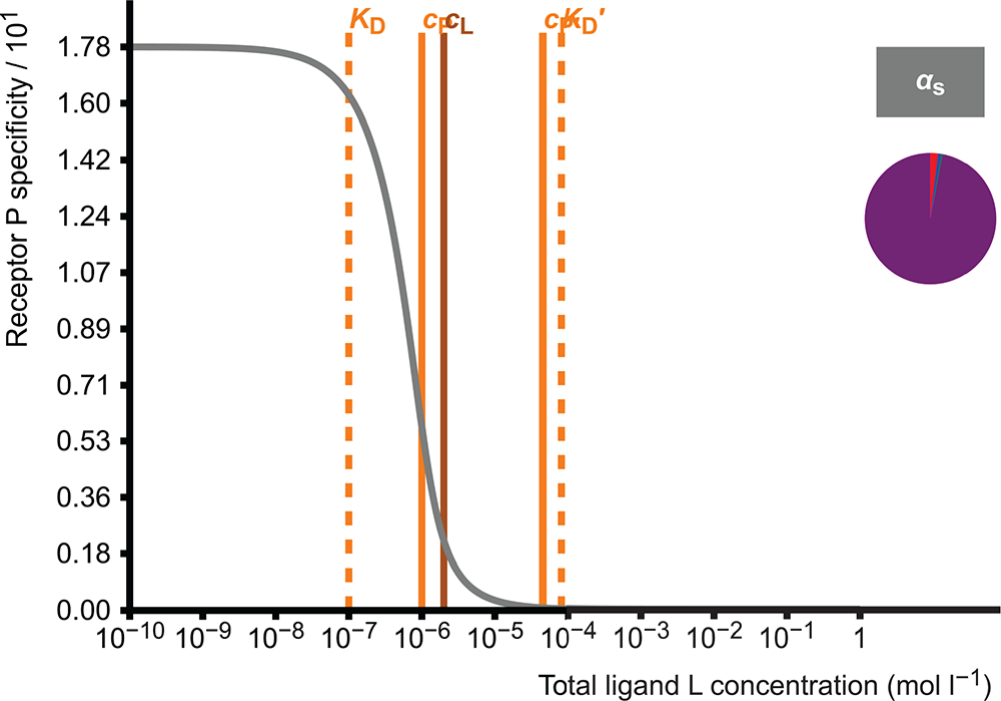
Specificity α_s_ as a function of ligand L concentration. The high affinity receptor (*K*_D_ = 1.0 × 10^–7^ mol L^–1^) has a concentration of *c*_P_ = 1.0 × 10^–6^ mol L^–1^. The low affinity receptor (*K*_D_^′^ = 8.0 × 10^–5^ mol L^–1^) has a concentration of *c*_P′_ = 4.5 × 10^–5^ mol L^–1^. The specificity gets the value 2.2 at the set value of *c*_L_ = 2.0 × 10^–6^ mol L^–1^.

The configuration resembles the preceding one, and the same thermodynamic laws apply. The ligand L binds competitively to two different receptors P and P′.
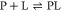
9
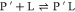
10There are five adjustable parameters in the model: three total concentrations (for two receptors and one ligand) and dissociation constants (*K*_D_ and *K*_D_^′^) for two complexes, which also produces a cubic equation. Equilibrium concentrations of all PL, P′L, P, P′, and L are calculated. The two receptors can be either in the same protein or in two different proteins. This model is useful in the analysis of ligand binding specificity as a function of ligand concentration and in the interpretation of binding site competition experiments.^6,7^ The model includes the calculation of the specificity factor
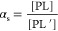
11as defined by Eaton et al.^8^ It shows higher values of specificity at low ligand concentration but decreases when the ligand concentration increases, indicating loss of specificity (Figure 4).

**Figure 5:**
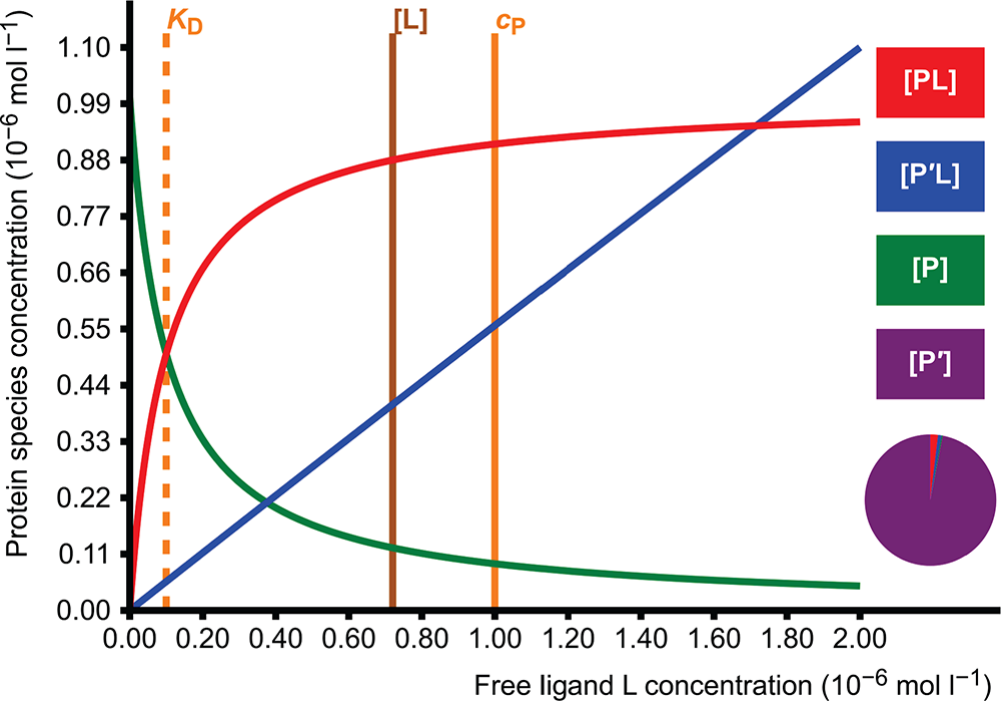
Ligand binding isotherms in the competing receptors simulation applet. The same parameters have been used as in Figure 4. The binding curve of the high-affinity receptor (P, red) is hyperbolic and nearly reaches the saturation limit, but the binding curve of the low-affinity receptor (P′, blue) appears to be linear in this concentration range. At the set value of *c*_L_ = 2.0 × 10^–6^ mol L^–1^, receptors P and P′ are 87.8% and 0.9% saturated, respectively, and the specificity α_s_ = 2.2. At [L] ≈ 1.7 × 10^–6^ mol L^–1^, the amount of low-affinity complexes P′L reaches the amount of high-affinity complexes PL.

The model can be also used in investigating the effect of nonspecific binding. The binding to a high-affinity receptor can be represented by low *K*_D_ and low receptor P concentration *c*_P_. The nonspecific binding can be considered to follow similar rules of binding but the affinity is much weaker and the number of binding sites are much higher. This nonspecific binding can be represented by using a high *K*_D_ and a high receptor P′ concentration *c*_P′_. Receptor P′ can be in the same protein or in a different protein. In this model, the binding curve for the high-affinity receptor P has a hyperbolic shape and a saturation limit. In principle, the binding curve for the low-affinity receptor would also have a saturation limit but at much higher ligand concentration. In the concentrations in which the binding curve for the binding to P shows a hyperbolic shape, the binding curve for P′ is approximately linear (Figure 5).

## 3 Results and Discussion

### 3.1 Simulation Applets

A set of four simulation applets have been developed for visualizing equilibrium concentrations. The applets have been written in HTML, JavaScript, and CSS code and implemented as web pages that run in a web browser. The mathematics have been worked out on paper and programmed as functions in JavaScript. All numbers are stored internally as double-precision floating-point numbers which are fast but suffer from rounding errors and loss of precision in some cases. However, as long as the adjustable parameters are kept in the ranges of the slider elements, these effects are insignificant and almost unnoticeable. The internal calculations use mostly simple arithmetics; cubic equations are solved using Newton’s method and the bisection method as fallback, and a least-squares fit algorithm has been written for the curve fitting. In addition to text and links, an applet page consists of input elements, an output graph, and an output table. Adjusting the input triggers JavaScript code to update the output. The graph is implemented as a Scalable Vector Graphics (SVG) object which can be zoomed in indefinitely in the browser and resized by dragging the bottom-right corner. The graph can also be exported as an SVG file by clicking the “Export graphic (SVG)” button, and the file can be opened in a graphics editor, for example in Inkscape,^9^ CorelDRAW,^10^ GIMP,^11^ or Photoshop.^12^ Screenshots of the user interfaces of each applet are shown in Figures S1–S4.

The simulation applets visualize the equilibria of the aforementioned reaction cases. The user is able to set total concentrations of the species and the dissociation constants of the complexes using sliders or, alternatively, manually by double-clicking the value label and editing the value in the appearing text box. In the latter case, any value in the range 10^–18^ to 10^3^ is allowed, though values outside the range of the sliders 10^–9^ to 10^–1^ may cause numerical and graphical glitches. This is indicated by the changing color of the text box: disallowed values are red, values in the range of the sliders are green, and others are dark yellow. The association constants *K*_A_ and the Gibbs free energies Δ*G* corresponding to the set dissociation constants are calculated and displayed as well. The *x*-axis is configurable to total concentrations or, in the homodimerization, ligand binding and competing receptors simulations, equilibrium concentrations [P], [L], and [L], respectively. The *y*-axis depicts either the absolute equilibrium concentrations of each species in a logarithmic scale or, in all but the competing receptors simulation, the relative amounts of the species in a linear scale. In the competing receptors simulation, instead of the relative amounts, absolute concentrations are shown in a linear scale, which was deemed to be a more useful and less confusing option. In addition to the curves, the applet shows the equilibrium concentrations at the specified point on the *x*-axis (the value of the parameter set as the *x*-axis) in a table under the graph and the proportional amounts as a pie diagram. All the adjustable parameters and calculated equilibrium concentrations in each applet are shown in Table 1.

**Table 1:** All the Adjustable Parameters and Calculated Equilibrium Concentrations in Each Simulation Applet^a^

	simulation applet			
	homodimerization	ligand binding	competing ligands	competing receptors
adjustable concentration parameters *c*	protein P	protein P	protein P	protein P
		ligand L	ligand L	protein P′
			ligand L′	ligand L
adjustable dissociation constants *K*_D_	dimer P_2_	complex PL	complex PL	complex PL
			complex PL′	complex P′L
calculated concentrations at equilibrium	monomer P	free protein P	free protein P	free protein P
	dimer P_2_	complex PL	complex PL	free protein P′
			complex PL′	complex PL
				complex P′L

a Less relevantly, in each case the masses of each protein and ligand can be input, which affects the equilibrium concentrations when expressed in mass units, and also for each dissociation constant, association constants and Gibbs free energies of association are displayed.

There is also a curve-fitting tool that can be used to approximate unknown initial concentrations or dissociation constants using data points. The interface is shown in Figure S5. The data are input in the text box as pairs of numbers row by row, expressed in plain text as decimal numbers or in the E notation (e.g., “0.00028” or “2.8e-4”). Once input, the data points are drawn in the graph as crosses. One can then choose any of the equilibrium concentration curves to be fitted to the data points. The unknown parameters must be set as free parameters by ticking the corresponding checkboxes, and the calculation attempts to find the values for them resulting in the best possible fit. There are three methods of finding the fit:

- **Two-pass search** first goes through the ranges of the sliders coarsely and picks the values where the sum of squared residuals is the smallest. Then, a fine search around that point is done to find the best possible solution. This is usually quick but can potentially converge to wrong results in extreme cases where the coarse search results in an incorrect point. This is the default option.
- **Single-pass search** simply does a full search of the whole input space, in other words, it checks all possible combinations of slider positions. This will always find the best possible solution lying in the defined intervals, but it is slow when more than two parameters are searched.
- **Iterative search** is like the second step of the two-step search, but the starting values (initial guess) are input with the sliders first, and steps are done iteratively until the search converges on a single point. If the initial guess is close to a solution (a local minimum of the sum of squared residuals), the search will find it quickly.

It is important to realize that the curve-fitting tool is not a proper analytical tool. Being constrained to the possible slider positions, it does not give the most accurate values nor, most importantly, uncertainties of any kind. More accurate estimates of *K*_D_ values can be obtained by nonlinear curve fitting (for example, in MATLAB^13^ or GNU Octave^14^) provided that an analytic solution for binding equilibria or an appropriate numerical fitting algorithm is available.

The applet source files are freely available via GitHub^15^ under the GNU General Public License, version 2.^16^ There is also a GitHub page^17^ (URL: https://protsim.github.io/protsim) with which the simulation pages can be directly accessed using a desktop or mobile web browser. The code can be freely downloaded, used, and modified by anyone provided that the authors are acknowledged by citing this paper and that any modifications contain all source code under the same or compatible license.

### 3.2 Examples of Use

#### 3.2.1 Planning a Measurement of Homodimerization Affinity

Consider a case in which the approximate dissociation constant of a protein dimer is known. For example, Haka et al.^18^ estimated a *K*_D_ of 0.8 mol L^–1^ for the Triple 3 variant of the Equ c 1 allergen using a single native mass spectrum. If the *K*_D_ was to be determined more accurately with multiple data points, at which protein concentrations should the new measurements be done? The method of measurement directly or indirectly returns the proportions of monomer and dimer at each tested total protein concentration.

**Figure 6:**
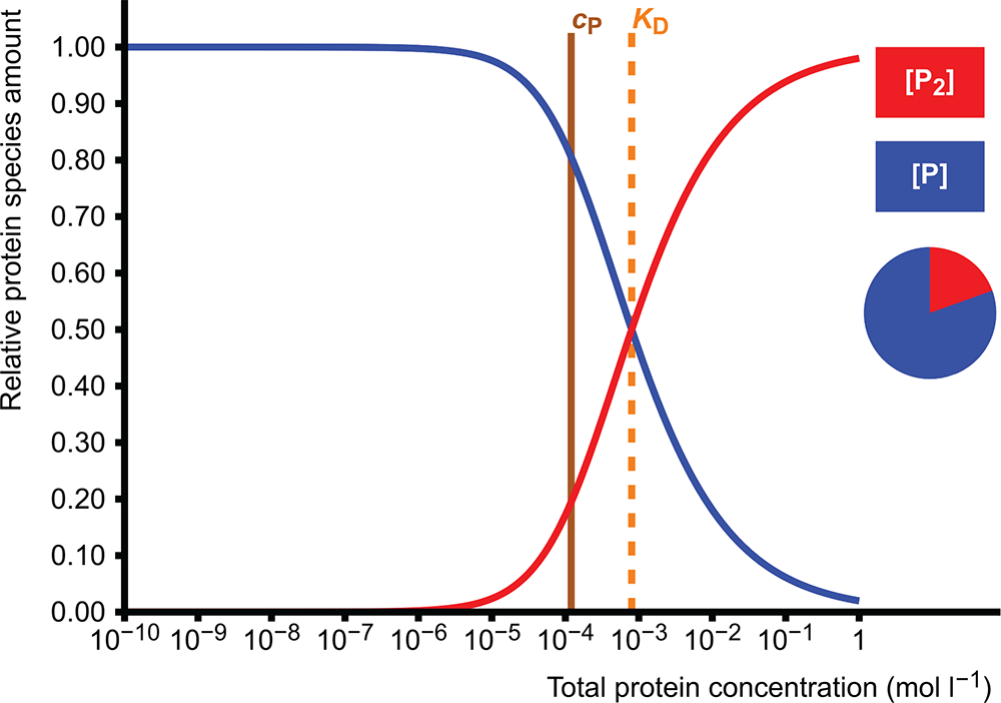
Relative amounts of protein monomer (P, blue) and dimer (P_2_, red) as concentrations of total protein concentration when the dissociation constant *K*_D_ = 8.0 × 10^–4^ mol L^–1^. At *c*_P_ = 1.2 × 10^–4^ mol L^–1^, the relative amounts of monomer and dimer are 80.5% and 19.5%, respectively.

In the protein homodimerization simulation, *K*_D_ is set to 8.0 × 10^–4^ mol L^–1^, vertical scale to relative protein concentration and horizontal axis to total protein concentration. The curves of [P_2_] and [P] show the behavior of the equilibrium when the total protein concentration changes. The range useful for the determination of *K*_D_ will be near the set value where both species are present in significant amounts. Say, if proportions less than 20% cannot be reliably observed, then it is not necessary to prepare and measure samples for which either proportion is less than 20%. When *c*_P_ is set to 1.2 × 10^–4^ mol L^–1^, the proportion of the dimer is 19.5% (Figure 6), and when it is set to 8.0 × 10^–3^ mol L^–1^, the proportion of the monomer is 20%. Therefore, the useful protein concentration range will be roughly 0.1–8 mmol L^–1^. Of course, if the initial estimate of *K*_D_ is inaccurate, the experiments may yield unexpected results, but this is the range worth checking first.

#### 3.2.2 Competitive Binding of Oxygen and Carbon Monoxide to Hemoglobin

**Figure 7:**
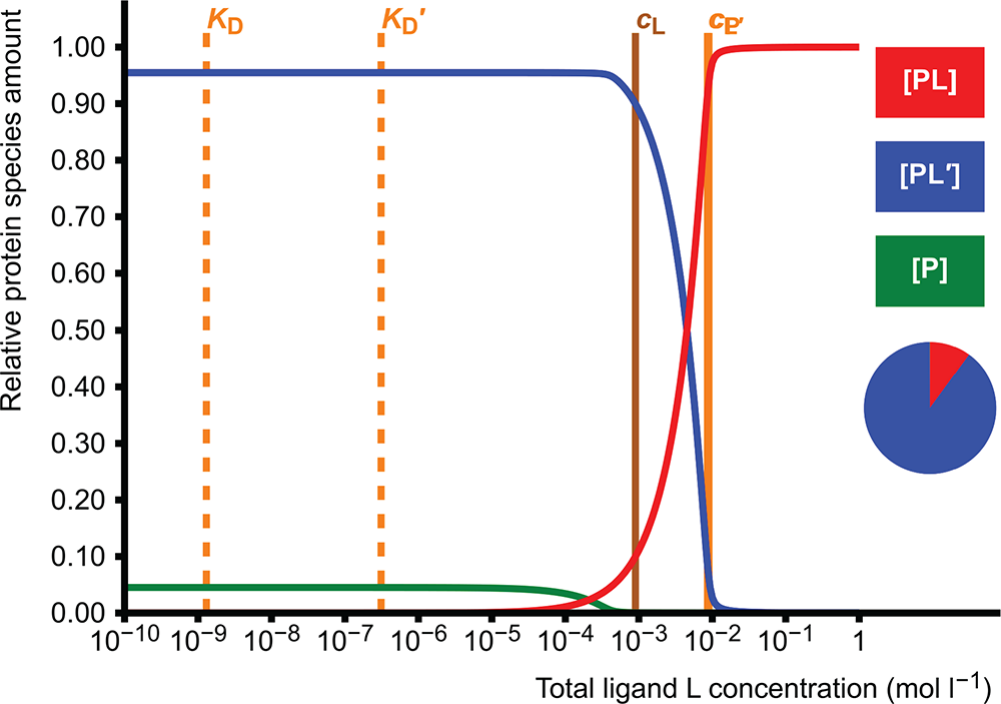
Competitive binding of two ligands with parameters *c*_P_ = 9.0 × 10^–3^ mol L^–1^, *c*_L_^′^ = 8.6 × 10^–3^ mol L^–1^, *K*_D_ = 1.3 × 10^–9^ mol L^–1^, and *K*_D_^′^ = 3.1 × 10^–7^ mol L^–1^, modeling the competitive binding of carbon monoxide (L) and oxygen (L′) to hemoglobin (P). When the total concentration of carbon monoxide (*c*_L_) increases to 9.0 × 10^–4^ mol L^–1^, the relative amount of carboxyhemoglobin (complex PL) is increased to 10%.

To demonstrate the competing ligands simulation, consider how hemoglobin can bind oxygen and carbon monoxide competitively with different affinities. In reality, hemoglobin exists as a homotetramer in biological conditions, and O_2_ or CO binding is associated with allosteric regulation (positive co-operativity). For simplicity, in this example, hemoglobin is treated as a monomeric protein with a single binding site for O_2_ or CO. The association constants of the complexes with carbon monoxide (L) and oxygen (L′) are *K*_D_ = 7.5 × 10^8^ L mol^–1^ and *K*_A_^′^ = 3.2 × 10^6^ L mol^–1^, respectively,^19^ and the corresponding dissociation constants are *K*_D_ = 1.3 × 10^–9^ mol L^–1^ and *K*_D_^′^ = 3.1 × 10^–7^ mol L^–1^, respectively. Let us use the value *c*_P_ = 9.0 × 10^–3^ mol L^–1^ (14.5 g/dl) for a normal hemoglobin concentration in human blood^20^ and set vertical scale to relative and horizontal axis to total ligand L concentration. First, to find out at which oxygen concentration the hemoglobin is 95% saturated (a healthy oxygen saturation), set *c*_L_ to the minimum slider value of 1.0 × 10^–9^ mol L^–1^ and find a value for *c*_L_^′^ such that the proportion of PL′ is 95%. This occurs roughly at *c*_L_^′^ = 8.6 × 10^–3^ mol L^–1^ (Figure 7). Then, increasing *c*_L_, the concentration of PL gradually increases and causes the proportion of PL′ to decrease. At *c*_L_ = 9.0 × 10^–4^ mol L^–1^ (10% of *c*_P_ and 10.5% of *c*_L_^′^), the proportion of PL is 10%, a level indicative of carbon monoxide poisoning.^21^ This shows how carbon monoxide displaces oxygen in hemoglobin when its concentration in blood increases, though in reality the situation is more complex: the dissolution of gases in blood is ignored, and because carbon monoxide tends to accumulate in the body, much lower concentrations in air than 10.5% of oxygen are sufficient for causing carbon monoxide poisoning over time.

#### 3.2.3 Dissociation Constant of Protein–Metal Complex

As an example, let us recreate the calculation of the dissociation constant of the xylonolactonase–iron complex reported by Pääkkönen et al.^5^ The raw data used in the original calculations are presented in Table 2. The data are the relative amounts of the complex against free iron concentration, so the vertical scale will be set as relative, and the horizontal axis will be set as the free ligand concentration. In this situation, the concentration of the protein *c*_P_ does not affect the shapes of the curves, but it can still be input as 1.2 × 10^–6^ mol L^–1^ using the slider. The setting of the free ligand concentration [L] only affects the pie diagram and the table of concentrations, so it can be set as any arbitrary value. The dissociation constant *K*_D_ can also be at any value at this point.

**Table 2:** Raw Data Used for the Determination of the Dissociation Constant of the Xylonolactonase–Iron Complex^a^

[L] (mol L^–1^)	*B*_PL_
1.70 × 10^–8^	0.0958
1.34 × 10^–8^	0.210
1.90 × 10^–7^	0.276
3.81 × 10^–7^	0.550
1.22 × 10^–6^	0.696
3.12 × 10^–6^	0.785
6.99 × 10^–6^	0.905
1.50 × 10^–5^	0.934

a The values are the relative amounts (*B*_PL_) of complex PL at varying free iron concentration [L]. The data have been reproduced with permission from the authors of the original publication.^5^ The same data are presented in copy-pastable form in the Supporting Information, section 4.1.

**Figure 8:**
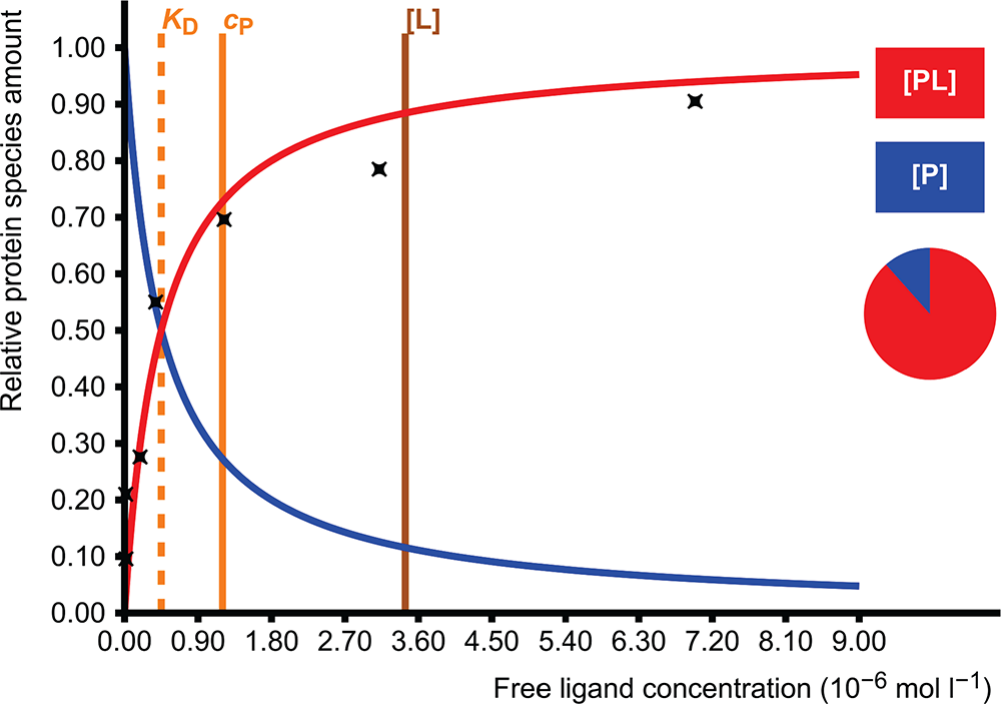
Least squares fit of the data in Table 2 in the ligand binding simulation. The concentration *c*_P_ is set as 1.2 × 10^–6^ mol L^–1^, and the dissociation constant *K*_D_ = 4.5 × 10^–7^ mol L^–1^ has been determined by the fitting algorithm. The pie diagram depicts the proportions of PL (88.4%) and P (11.6%) at the set value of *c*_L_ = 10*K*_D_ = 4.5 × 10^–6^ mol L^–1^.

The choice of the calculation method does not matter in this case since they will all give the correct answer reasonably fast. When the curve [PL] is set to be calculated, *K*_D_ is chosen as the free parameter, and “Calculate” is clicked, the algorithm finds the least-squares fit and sets the *K*_D_ as 4.5 × 10^–7^ mol L^–1^. The corresponding values of the association constant *K*_A_ = 2.2 × 10^6^ L mol^–1^ and the Gibbs free energy Δ*G* = −36.2 kJ mol^–1^ (*T* = 25 °C) are also displayed. The resulting graph, when *c*_L_ is set as 10*K*_D_ = 4.5 × 10^–6^ mol L^–1^, is shown in Figure 8.

The reported value for *K*_D_, calculated using unweighted orthogonal distance regression, is (5.0 ± 1.3) × 10^–7^ mol L^–1^. The values are different because the regression methods are different and because the model in the publication^5^ also accounts for the maximum saturation level, which this model assumes as unity. In any case, this result would be a reasonable approximation of the correct value of *K*_D_, though only to the precision of one significant digit despite the applet displaying two.

#### 3.2.4 Dissociation Constant of Protein Dimer

As another example, let us recreate the calculation of the dissociation constant of the wild-type Equ c 1 allergen dimer reported by Haka et al.^18^ The raw data used in the original calculations are presented in Table 3. The data are the concentrations of free monomer [P] against total protein concentration *c*_P_, so the vertical scale will be absolute, and the horizontal axis will be total protein concentration. The values of *c*_P_ and *K*_D_ can be set as any arbitrary value at this point.

**Table 3:** Raw Data Used for the Determination of the Dissociation Constant of the Equ c 1 Allergen Dimer^a^

*c*_P_ (mol L^–1^)	[P] (mol L^–1^)
6.2 × 10^–8^	9.74 × 10^–9^
4.36 × 10^–7^	4.71 × 10^–8^
7.47 × 10^–7^	8.21 × 10^–8^
1.81 × 10^–6^	1.19 × 10^–7^
2.55 × 10^–6^	1.29 × 10^–7^
3.68 × 10^–6^	1.74 × 10^–7^
4.73 × 10^–6^	1.84 × 10^–7^

a The values are the free monomer concentrations [P] at varying total protein concentration *c*_P_. The data have been reproduced with permission from the authors of the original publication.^18^ The same data are presented in copy-pastable form in the Supporting Information, section 4.2.

**Figure 9:**
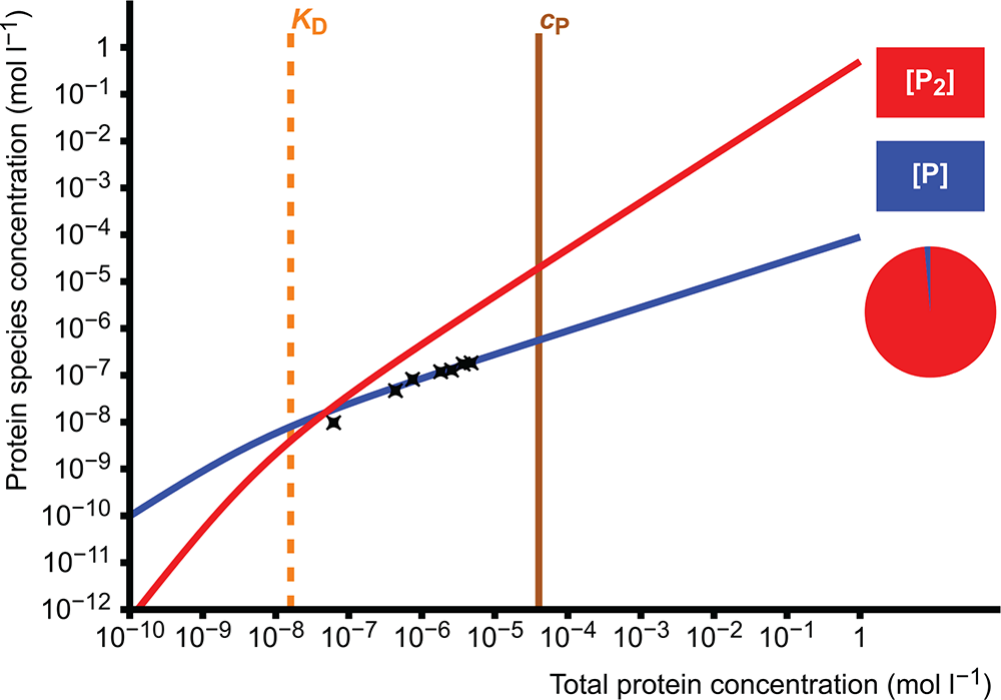
Least squares fit of the data in Table 3 in the homodimerization simulation. The dissociation constant *K*_D_ = 1.6 × 10^–8^ mol L^–1^ has been determined by the fitting algorithm. The pie diagram depicts the proportions of P_2_ (98.6%) and P (1.4%) at the set value of *c*_P_ = 4.0 × 10^–5^ mol L^–1^.

The data are input as described above, and the calculation method can be left as the default value. When the curve [P] is set to be calculated and “Calculate” is clicked, the calculation yields *K*_D_ = 1.6 × 10^–8^ mol L^–1^, *K*_A_ = 6.2 × 10^7^ L mol^–1^ and Δ*G* = −44.5 kJ mol^–1^. The resulting graph, when *c*_P_ is set as 4.0 × 10^–5^ mol L^–1^ (as in the measurement of the mass spectrum in Figure 2B of the publication^18^), is shown in Figure 9. The reported value for the *K*_D_ is (1.56 ± 0.08) × 10^–8^ mol L^–1^, very close to this result. The authors have done a similar least-squares fit, and this calculation would have converged to the same value if it were not restricted to the discrete values of the slider.

## 4 Conclusions

The presented simulation applets are useful for visualizing the behavior of equilibrium reactions as shown in all figures and the examples of use. As demonstrated, the applets can be used for planning experiments, for predicting the behavior of systems to which they are applicable and for estimating unknown parameters based on experimental data. Similarly, experimental results can be verified by calculating the theoretical behavior and comparing it to the experimental behavior. Anyone with sufficient programming skills can download the applets and modify them according to their needs and preferences. The hope is that these applets will be useful for researchers who work with equilibrium reactions like these. In the referenced works, such tools have not been available, and while alternative methods for calculation and visualization are perfectly valid, using these simple applets would speed up the workflow and reduce errors.

## Supporting Information Available

Detailed mathematical models, data of the curve-fitting examples in copy-pastable format, figures of applet interfaces (PDF)
