# Supplementary material for: Calculation and Visualization of Binding Equilibria in Protein Studies
Source: ACS Omega. 2022 Mar 16;7(12):10789–95. doi: 10.1021/acsomega.2c00560 (PMC8973030; doi:10.1021/acsomega.2c00560)
Supplement: Supplementary file 1 — ao2c00560_si_001.pdf [file ao2c00560_si_001.pdf]

Supporting Information

Calculation and Visualization of Binding Equilibria in Protein Studies

Johan Pääkkönen*, Janne Jänis*, and Juha Rouvinen*†

* Department of Chemistry, University of Eastern Finland, P.O. Box 111, 80101 Joensuu, Finland

† Corresponding author – e-mail: juha.rouvinen@uef.fi

Table of Contents

1 Homodimerisation (Self-Association) S2

1.1 As a Function of Free Protein Concentration S2

1.2 As a Function of Total Protein Concentration S2

2 Heterodimerisation (Ligand Binding to a Receptor) S3

2.1 As a Function of Free Ligand Concentration S3

2.2 As a Function of Total Ligand Concentration S3

3 Competitive Binding of a Ligand to Two Receptors S4

3.1 As a Function of Free Ligand Concentration S4

3.2 As a Function of Total Ligand Concentration S4

3.3 Competitive Binding of Two Ligands to a Receptor S5

4 Copy-Pastable Data of the Curve Fitting Examples S5

4.1 Dissociation Constant of Protein–Metal Complex (Table 2) S6

4.2 Dissociation Constant of Protein Dimer (Table 3) S6

5 Figures of Applet Interfaces S7

1 Homodimerisation (Self-Association)

Reaction equation:

$$\mathrm{P} + \mathrm{P} \rightleftharpoons \mathrm{P}_2 \quad (K_\mathrm{D}) \tag{1}$$

Total concentration and dissociation constant:

$$c_\mathrm{P} = [\mathrm{P}] + 2[\mathrm{P}_2] \tag{2}$$

$$K_\mathrm{D} = [\mathrm{P}]^2/[\mathrm{P}_2] \tag{3}$$

1.1 As a Function of Free Protein Concentration

If the free protein concentration [P] and the dissociation constant K_D are known, the concentration of the dimer $[\mathrm{P}_2]$ is calculated as

$$[\mathrm{P}_2] = \frac{[\mathrm{P}]^2}{K_\mathrm{D}}. \tag{4}$$

Alternatively, the proportional amounts of monomer B_P and dimer B_{P_2} can be calculated. These formulae are sometimes used in determination of K_D: if, for instance, B_P is known at different values of [P], fitting Equation (5) to the data yields K_D.

$$B_\mathrm{P} \equiv \frac{[\mathrm{P}]}{c_\mathrm{P}} = \frac{K_\mathrm{D}}{2[\mathrm{P}] + K_\mathrm{D}} \tag{5}$$

$$B_{\mathrm{P}_2} \equiv \frac{2[\mathrm{P}_2]}{c_\mathrm{P}} = \frac{2[\mathrm{P}]}{2[\mathrm{P}] + K_\mathrm{D}} \tag{6}$$

1.2 As a Function of Total Protein Concentration

If the total protein concentration c_P is known instead of the free protein concentration [P], [P] is calculated by solving the quadratic equation

$$2[\mathrm{P}]^2 + K_\mathrm{D}[\mathrm{P}] - c_\mathrm{P}K_\mathrm{D} = 0. \tag{7}$$

The equation has two roots, but only the greater one is positive, so the relevant solution is

$$[\mathrm{P}] = \frac{1}{4}\left(-K_\mathrm{D} + \sqrt{K_\mathrm{D}^2 + 8c_\mathrm{P}K_\mathrm{D}}\right), \tag{8}$$

and $[\mathrm{P}_2]$ is calculated with Equation (4).

2 Heterodimerisation (Ligand Binding to a Receptor)

Reaction equation:

$$\mathrm{P} + \mathrm{L} \rightleftharpoons \mathrm{PL} \quad (K_\mathrm{D}) \tag{9}$$

Total concentrations and dissociation constant:

$$c_\mathrm{P} = [\mathrm{P}] + [\mathrm{PL}] \tag{10}$$
$$c_\mathrm{L} = [\mathrm{L}] + [\mathrm{PL}] \tag{11}$$
$$K_\mathrm{D} = [\mathrm{P}][\mathrm{L}]/[\mathrm{PL}] \tag{12}$$

2.1 As a Function of Free Ligand Concentration

If the concentration of the free ligand [L], total protein concentration c_P and the dissociation constant K_D are known, the proportions of free protein B_P and complex B_PL are written as:

$$B_\mathrm{P} \equiv \frac{[\mathrm{P}]}{c_\mathrm{P}} = \frac{K_\mathrm{D}}{[\mathrm{L}] + K_\mathrm{D}}, \tag{13}$$
$$B_\mathrm{PL} \equiv \frac{[\mathrm{PL}]}{c_\mathrm{P}} = \frac{[\mathrm{L}]}{[\mathrm{L}] + K_\mathrm{D}}. \tag{14}$$

The other equilibrium concentrations are calculated as:

$$[\mathrm{P}] = c_\mathrm{P} B_\mathrm{P} = \frac{c_\mathrm{P} K_\mathrm{D}}{[\mathrm{L}] + K_\mathrm{D}}, \tag{15}$$
$$[\mathrm{PL}] = c_\mathrm{P} B_\mathrm{PL} = \frac{c_\mathrm{P}[\mathrm{L}]}{[\mathrm{L}] + K_\mathrm{D}} = c_\mathrm{P} - [\mathrm{P}]. \tag{16}$$

2.2 As a Function of Total Ligand Concentration

If the total ligand concentration c_L is known instead of the concentration of free ligand [L], [L] is calculated by solving the quadratic equation

$$[\mathrm{L}]^2 - (c_\mathrm{L} - c_\mathrm{P} - K_\mathrm{D})\,[\mathrm{L}] - c_\mathrm{L} K_\mathrm{D} = 0. \tag{17}$$

The equation has two roots, but only the greater one is positive, so the relevant solution is

$$[\mathrm{L}] = \frac{1}{2}\left(c_\mathrm{L} - c_\mathrm{P} - K_\mathrm{D} + \sqrt{(c_\mathrm{L} - c_\mathrm{P} - K_\mathrm{D})^2 + 4 c_\mathrm{L} K_\mathrm{D}}\right), \tag{18}$$

and the other equilibrium concentrations are calculated with Equations (15) and (16). The applet does the calculations in a different but equivalent way that is numerically stable.

3 Competitive Binding of a Ligand to Two Receptors

Reaction equations:

$$\mathrm{P} + \mathrm{L} \rightleftharpoons \mathrm{PL} \quad (K_\mathrm{D}) \tag{19}$$

$$\mathrm{P}' + \mathrm{L} \rightleftharpoons \mathrm{P}'\mathrm{L} \quad (K'_\mathrm{D}) \tag{20}$$

Total concentrations and dissociation constants:

$$c_\mathrm{P} = [\mathrm{P}] + [\mathrm{PL}] \tag{21}$$

$$c_{\mathrm{P}'} = [\mathrm{P}'] + [\mathrm{P}'\mathrm{L}] \tag{22}$$

$$c_\mathrm{L} = [\mathrm{L}] + [\mathrm{PL}] + [\mathrm{P}'\mathrm{L}] \tag{23}$$

$$K_\mathrm{D} = [\mathrm{P}][\mathrm{L}]/[\mathrm{PL}] \tag{24}$$

$$K'_\mathrm{D} = [\mathrm{P}'][\mathrm{L}]/[\mathrm{P}'\mathrm{L}] \tag{25}$$

3.1 As a Function of Free Ligand Concentration

If the free ligand concentration [L], the concentrations c_P and $c_{\mathrm{P}'}$ and the dissociation constants K_D and K'_D are known, the bindings to both receptors are considered separately and calculated as in Section 2.1. That is to say, Equations (15) and (16) yield the concentrations [P] and [PL] respectively and, when c_P and K_D are replaced by $c_{\mathrm{P}'}$ and K'_D respectively, concentrations [P′] and [P′L].

$$[\mathrm{P}] = \frac{c_\mathrm{P} K_\mathrm{D}}{[\mathrm{L}] + K_\mathrm{D}} \tag{26}$$

$$[\mathrm{PL}] = \frac{c_\mathrm{P}[\mathrm{L}]}{[\mathrm{L}] + K_\mathrm{D}} = c_\mathrm{P} - [\mathrm{P}] \tag{27}$$

$$[\mathrm{P}'] = \frac{c_{\mathrm{P}'} K'_\mathrm{D}}{[\mathrm{L}] + K'_\mathrm{D}} \tag{28}$$

$$[\mathrm{P}'\mathrm{L}] = \frac{c_{\mathrm{P}'}[\mathrm{L}]}{[\mathrm{L}] + K'_\mathrm{D}} = c_{\mathrm{P}'} - [\mathrm{P}'] \tag{29}$$

3.2 As a Function of Total Ligand Concentration

If the total ligand concentration c_L is known instead of the concentration of free ligand [L], [L] must be solved from the system of equations. It turns out that it requires solving the cubic equation

$$[\mathrm{L}]^3 + p[\mathrm{L}]^2 + q[\mathrm{L}] + r = 0 \tag{30}$$

where

$$p = c_{\mathrm{P}} + c_{\mathrm{P'}} - c_{\mathrm{L}} + K_{\mathrm{D}} + K'_{\mathrm{D}}, \tag{31}$$

$$q = c_{\mathrm{P}} K'_{\mathrm{D}} + c_{\mathrm{P'}} K_{\mathrm{D}} + K'_{\mathrm{D}} K_{\mathrm{D}} - c_{\mathrm{L}} \left(K_{\mathrm{D}} + K'_{\mathrm{D}} \right), \tag{32}$$

$$r = -c_{\mathrm{L}} K_{\mathrm{D}} K'_{\mathrm{D}}. \tag{33}$$

The equation is difficult to solve analytically, so the root in the range $0 < [\mathrm{L}] < c_{\mathrm{L}}$ is found numerically using Newton's method or, if it fails to converge within a set number of iterations, the bisection method. Albeit slow, it is guaranteed to converge to the solution since there will always be a single solution between zero and initial ligand concentration. Thereafter, the other equilibrium concentrations are calculated with Equations (26), (27), (28) and (29).

3.3 Competitive Binding of Two Ligands to a Receptor

Reaction equations:

$$\mathrm{P} + \mathrm{L} \rightleftharpoons \mathrm{PL} \quad (K_{\mathrm{D}}) \tag{34}$$

$$\mathrm{P} + \mathrm{L'} \rightleftharpoons \mathrm{PL'} \quad (K'_{\mathrm{D}}) \tag{35}$$

All the laws presented previously in Sections 3.1 and 3.2 apply in this case, only P, P′ and L are substituted by L, L′ and P respectively.

4 Copy-Pastable Data of the Curve Fitting Examples

These lists of numbers can be copy-pasted for recreating the curve fitting examples in the article. They contain the same data as Tables 1 and 2 but in a format that can be copy-pasted into the text box. The header rows do not need be copied, but if they are copied, the applet ignores them and no ill effects will occur. See the table captions for explanations of the values.

4.1 Dissociation Constant of Protein–Metal Complex (Table 2)

[L] ($mol\ l^{-1}$)	B_{PL}
1.70e-8	0.0958
1.34e-8	0.210
1.90e-7	0.276
3.81e-7	0.550
1.22e-6	0.696
3.12e-6	0.785
6.99e-6	0.905
1.50e-5	0.934

4.2 Dissociation Constant of Protein Dimer (Table 3)

c_P ($mol\ l^{-1}$)	[P] ($mol\ l^{-1}$)
6.20e-8	9.74e-9
4.36e-7	4.71e-8
7.47e-7	8.21e-8
1.81e-6	1.19e-7
2.55e-6	1.29e-7
3.68e-6	1.74e-7
4.73e-6	1.84e-7

5 Figures of Applet Interfaces

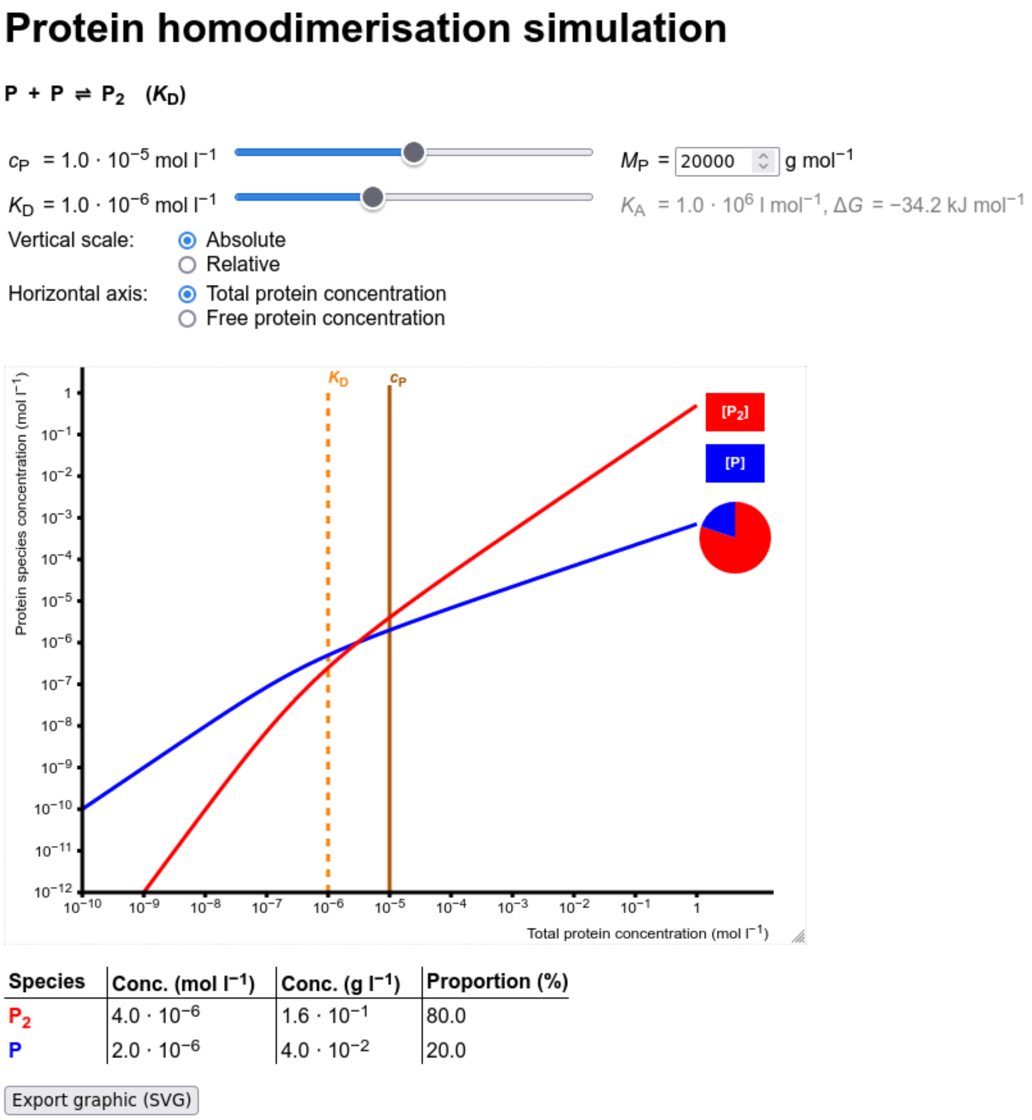

Figure S1. The user interface of the homodimerisation simulation applet. All settings are at default. In this and the following ones, the appearance is dependent on the web browser and installed fonts, so the interfaces may look different on any individual device.

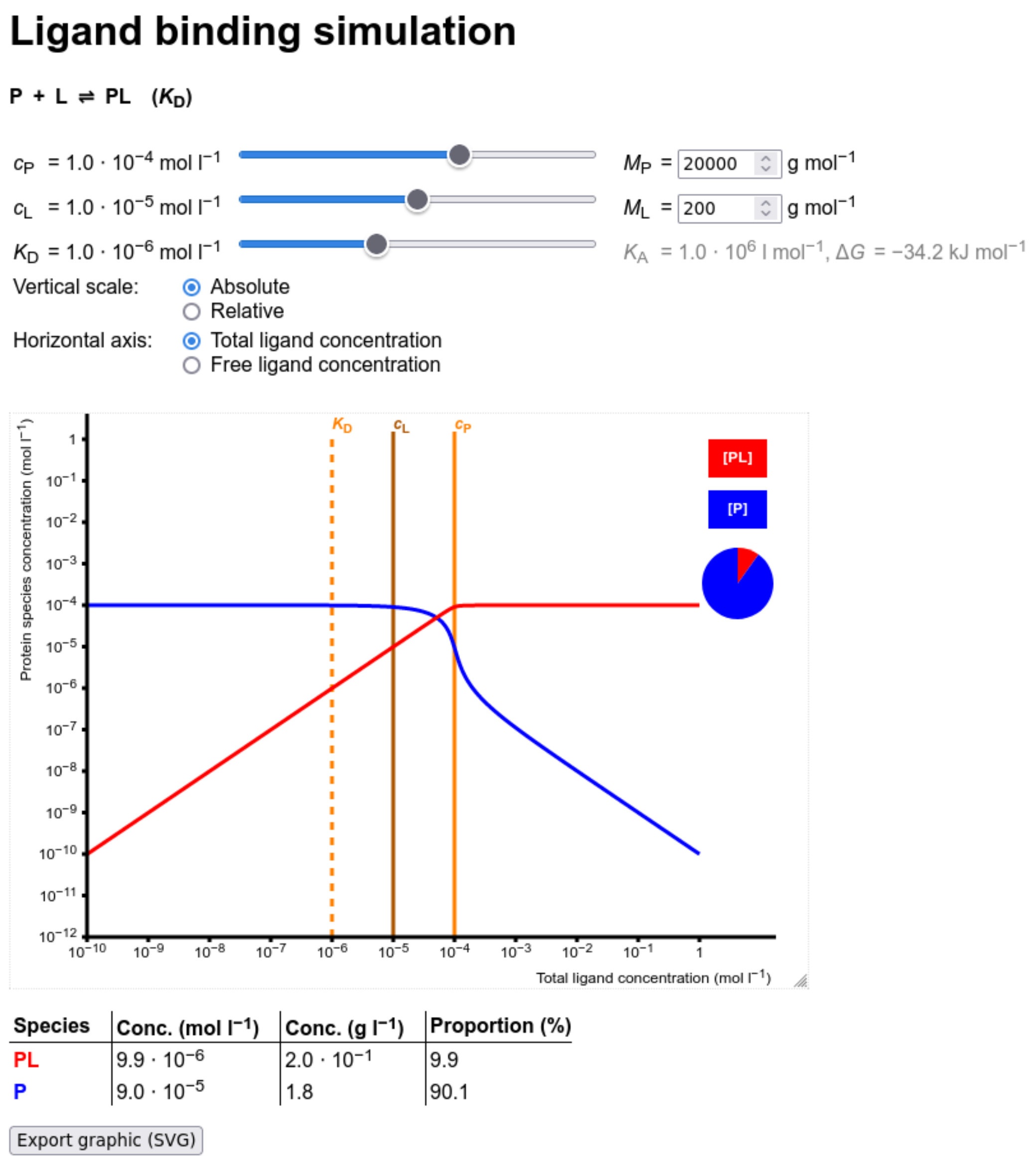

Figure S2. The user interface of the ligand binding simulation applet. All settings are at default.

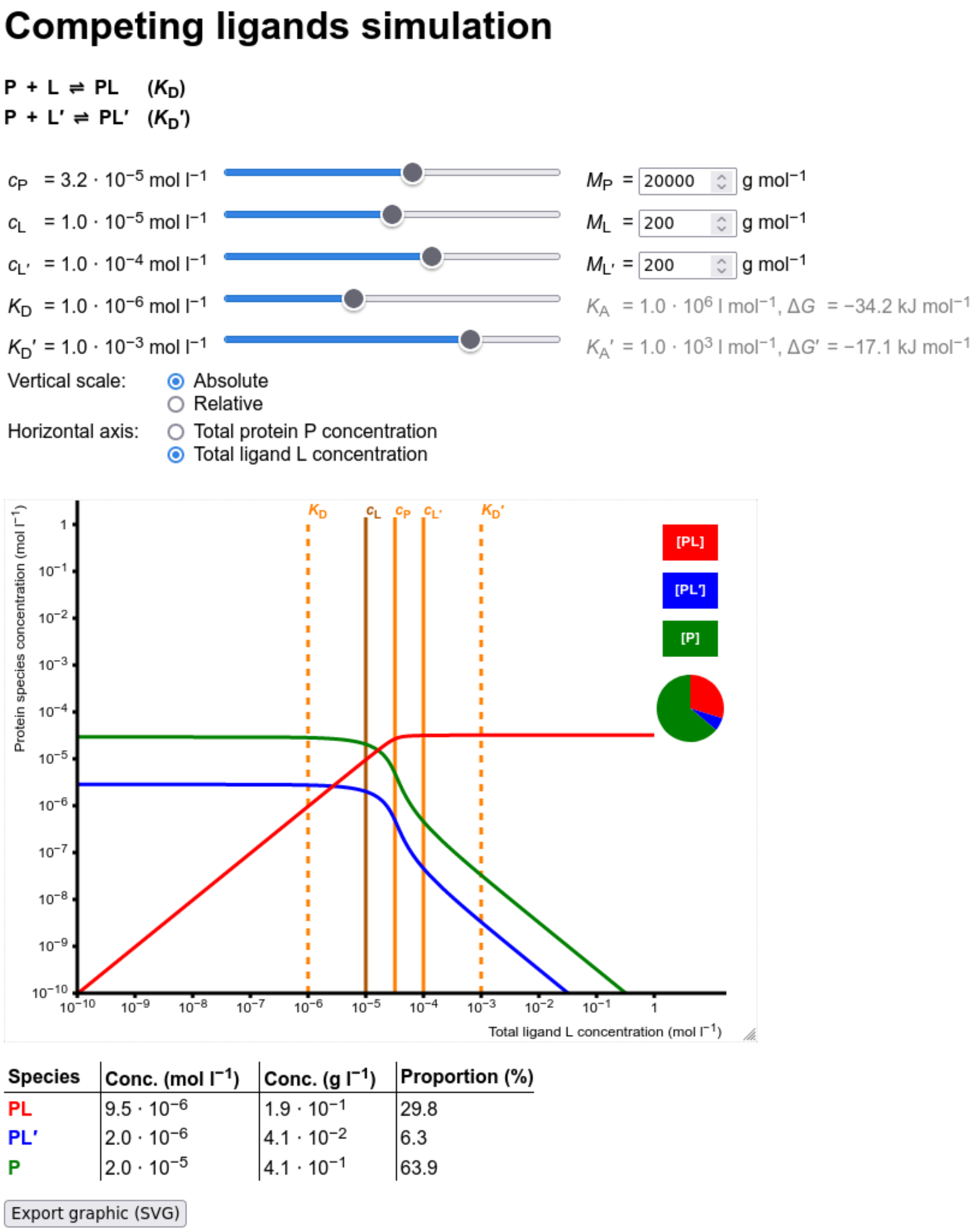

Figure S3. The user interface of the competing ligands simulation applet. All settings are at default.

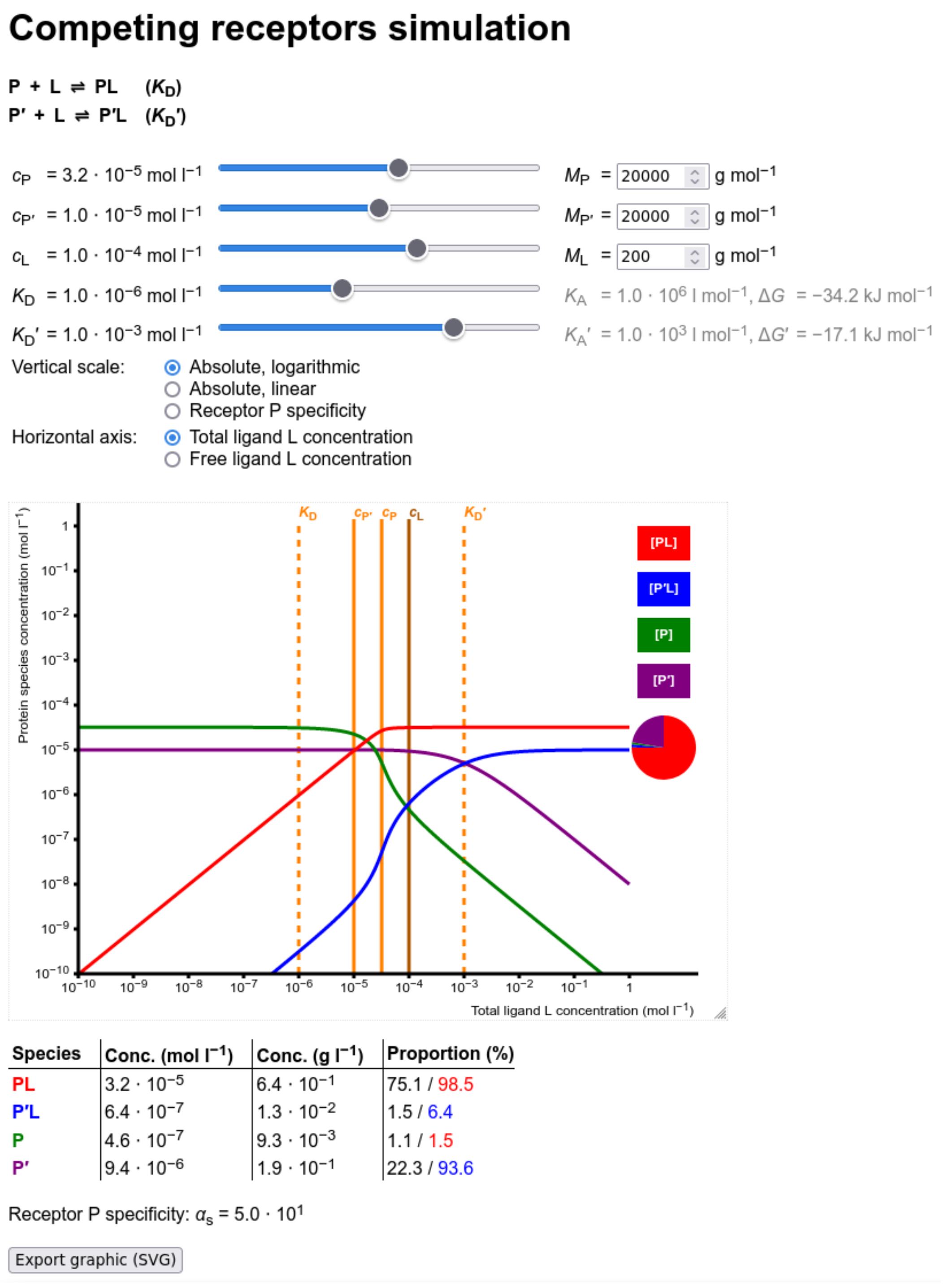

Figure S4. The user interface of the competing receptors simulation applet. All settings are at default.

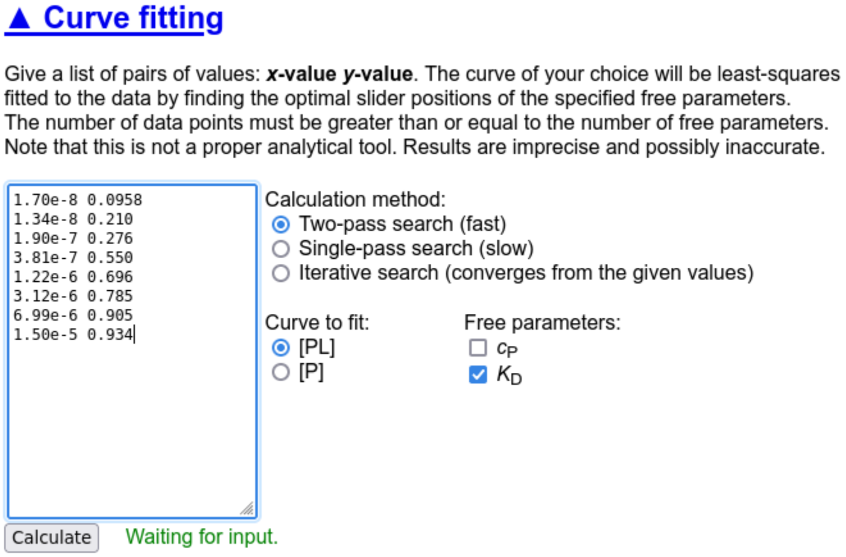

Figure S5. The user interface of the curve fitting tool in the ligand binding simulation applet. The entered data are the values used in the "Dissociation constant of protein–metal complex" example.
